# UV–Vis and ATR–FTIR spectroscopic investigations of postmortem interval based on the changes in rabbit plasma

**DOI:** 10.1371/journal.pone.0182161

**Published:** 2017-07-28

**Authors:** Qi Wang, Haijun He, Bing Li, Hancheng Lin, Yinming Zhang, Ji Zhang, Zhenyuan Wang

**Affiliations:** Department of Forensic Pathology, College of Forensic Medicine, Xi’an Jiaotong University, Xi’an, Shaanxi, China; Gaziosmanpasa Universitesi, TURKEY

## Abstract

Estimating PMI is of great importance in forensic investigations. Although many methods are used to estimate the PMI, a few investigations focus on the postmortem redistribution. In this study, ultraviolet–visible (UV–Vis) measurement combined with visual inspection indicated a regular diffusion of hemoglobin into plasma after death showing the redistribution of postmortem components in blood. Thereafter, attenuated total reflection–Fourier transform infrared (ATR–FTIR) spectroscopy was used to confirm the variations caused by this phenomenon. First, full-spectrum partial least-squares (PLS) and genetic algorithm combined with PLS (GA-PLS) models were constructed to predict the PMI. The performance of GA-PLS model was better than that of full-spectrum PLS model based on its root mean square error (RMSE) of cross-validation of 3.46 h (*R*^*2*^ = 0.95) and the RMSE of prediction of 3.46 h (*R*^*2*^ = 0.94). The investigation on the similarity of spectra between blood plasma and formed elements also supported the role of redistribution of components in spectral changes in postmortem plasma. These results demonstrated that ATR-FTIR spectroscopy coupled with the advanced mathematical methods could serve as a convenient and reliable tool to study the redistribution of postmortem components and estimate the PMI.

## Introduction

Determining the postmortem interval (PMI) is a task of great importance in daily forensic casework. An accurate determination of the PMI is of great forensic value for the crime scene investigator in driving investigation direction, setting terms of reference for an alibi, or pinpointing a suspect. Progress has been made in exploring new methods focused on the changes in biomolecules or other regular morphometric, physical, and chemical indices[1–7], excluding some traditional methods to estimate PMI, such as the evaluation of cooling of the body, rigor mortis, stomach contents, livor mortis, and insect growth after death [8, 9]. Conversely, most of these exploratory methods either require sophisticated procedures or are destructive to the limited forensic sample. Therefore, implementing a quick nondestructive and sample-saving method would be highly advantageous for determining the PMI at a crime scene.

Ultraviolet–visible (UV–Vis) spectroscopy is a quantitative analysis tool that can be applied as a reliable biosensor for detecting hemolysis in plasma and serum samples based on the specific absorption spectrum of hemoglobin (in particular, on oxyhemoglobin’s absorbance peak at 414 nm) [10, 11]. The attenuated total reflectance–Fourier transform infrared (ATR–FTIR) technique is a highly sensitive analytical tool widely used to detect the changes in the functional groups, bonding type, and molecular conformations of biochemical composition, such as nucleic acids, carbohydrates, proteins, and lipids [12, 13]. Unlike UV-Vis spectroscopy, nearly every biological molecule absorbs mid-infrared light, leading to characteristic spectra with multiple distinct peaks [14]. Thus, ATR-FTIR spectroscopy could provide more spectral information and be more favorable to distinguish the postmortem spectral variations of different samples. With the characteristics of minimal sample consumption and low cost, the measurement of the mid-infrared spectrum through ATR is also a direct and nondestructive way to extract original and plentiful spectral information of samples in a short time [15]. Now, portable and handheld FTIR instruments are commercially available [16, 17] and may be used for determining the PMI *in situ* at a crime scene in the future. These characteristics make ATR-FTIR spectroscopy go especially well with the forensic need for fast and precise determination of PMI.

Blood is seldom affected by confounding factors such as age, gender, diet, diurnal cycles, and stress thus making it an ideal candidate to reduce individual differences [18]. Therefore, it has been used to estimate PMI by measuring different candidate variables [1, 19–23]. As a liquid component of blood, plasma is one of the most common body fluids in the forensic lab analysis that can provide pathological and toxicological information of the victims, and is easily accessible even without performing a forensic autopsy. Another advantage is that complicated pretreatments such as homogeneity, freeze-drying, or mixing with KBr are not necessary for the ATR–FTIR and UV–Vis analyses of the plasma.

The chemical analysis of the plasma using UV–Vis and ATR–FTIR measurements may be a quick and easy way to provide insights into the postmortem biochemical changes. Previous studies primarily focused on some substantial organs from humans and animals, and the results showed that their FTIR spectra changes were highly correlated with PMI [24–27]. The aim of the present study was to obtain a complete overview of postmortem changes in blood using UV–Vis and ATR–FTIR spectroscopies and build more accurate and reliable mathematical models to determine PMI.

## Materials and methods

### Animal specimens and sample preparation

Male New Zealand White rabbits (*n* = 96, weight 2.2–2.5 kg), purchased from the Animal Center of Xi’an Jiaotong University, were anesthetized by urethane through abdomen, and then were sacrificed by air embolism at one of the auricular veins. No sample was collected prior to sacrifice. All of the animal experiments in the present study were specifically approved and overseen by the Care and Use of Laboratory Animal Committee of Xi’an Jiaotong University. Cadavers were kept at moderate ambient temperatures (*T*_a_, 25 ± 1°C) and relative humidity (RH, 40% ± 5%) in a controlled-environment chamber following sacrifice. For calibration set, arterial blood samples from 72 rabbits were taken at 0, 6, 12, 18, 24, 30, 36, 42, and 48 h (8 samples for each time point) after death. The arterial blood samples of other 24 rabbits used as a prediction set were taken at the postmortem time points of 3, 9, 15, 21, 27, 33, 39, and 45 h (3 samples for each time point). All arterial blood samples were collected from the left ventricle into tubes containing anticoagulant dipotassium ethylenediaminetetraacetic acid (K_2_EDTA). All samples were centrifuged at 4°C, 3000 rpm, for 15 min. Then, 20 μL of the supernatant plasma was collected into a 200-μL microcentrifuge tube. Deposits exclusively from 0-h samples were subsequently washed five times with 0.9% (w/v) NaCl, and the buffy coat was remained carefully each time to obtain 0-h formed elements of arterial blood. All samples were kept frozen at –80°C until tested.

### UV-Vis spectroscopy

A total of 1 μL of plasma was added to 200 μL of 0.01% Na_2_CO_3_. After sufficient mixing, UV–Vis absorbance was measured at 414 nm using the NanoDrop 2000 Spectrophotometer (Thermo Scientific, MA, USA) by applying 2 μL of the sample on the microvolume pedestal.

### ATR–FTIR measurements

A Thermo Nicolet-5700 FTIR spectrometer (Thermo Electron Scientific Instruments Corp., WI, USA) equipped with a diamond ATR accessory, a deuterated triglycine sulfate detector, and a KBr beam splitter was used for spectral acquisition. Infrared spectra analysis software package OMNIC version 8.2 (Thermo Nicolet Analytical Instruments, WI, USA) was used for analyzing the FTIR spectra and recording the data from the spectra. Three 1-μL subsamples of each sample were placed on the diamond ATR crystal and air-dried completely using a blow dryer to form homogeneous dried films. For each subsample, three replicate spectra were recorded to ensure the spectral reproducibility and assess analytical precision. All spectra were recorded in the range of 4000–900 cm^-1^ using the ATR method with a resolution of 4 cm^–1^ and 32 scans.

### ATR–FTIR spectral pretreatment

In the dataset treatment, the average of nonuplicate spectra of each sample was used. The interest was in researching postmortem changes in the “bio-fingerprint” region, which contained the fundamental vibrational energy absorbing frequencies of many biomolecules [28]. Therefore, only the spectral region between 1800 and 900 cm^–1^ was used for further analysis. Many preprocessing techniques were developed for raw spectra correction, including standard normal variate (SNV), multiplicative scatter correction, autoscaling, mean centering, and so on, to improve the robustness and accuracy of subsequent multivariate analyses. Moreover, the second-derivative spectrum served as a widely used preprocessing method in spectroscopic analysis. The negative peaks corresponded to the center of absorbance peaks of nonderivative spectrum. The method could not only remove baseline and linear trend but also allowed detection and positive identification of overlapping bands, leading to the improvement in both qualitative and quantitative perspectives in principle [29, 30]. The second-derivative peaks separated and observed in the amide region could also reflect secondary structure composition of proteins, and the relative intensities of these peaks were thereby considered to be related to the original intensity [30]. The spectra were corrected by SNV and subsequently converted into second derivatives using a 7-point Savitsky–Golay second-derivative function after checking several alternatives.

Two samples were classed as outliers and removed based on the leverage values, Q-residuals, and Studentized y-residuals [31]. Finally, only 70 samples of the calibration set were included for variable selection and model development.

### Partial least-squares regression

As a recently developed generalization of multiple linear regression, partial least-squares (PLS) regression is one of the most widely used modeling approaches for high-throughput data, particularly for quantitative analysis purposes. The general purpose of PLS is to handle regression problem by extracting these latent variables (LVs) and modeling the linear relationship between a set of explanatory variables *X* (*n*, *p*) and a response *y* (*n*, 1) [32, 33], such as the spectral data and the PMI values in this case. For PLS algorithm, the determination of LV number is a critical step which can optimize the predictive ability of the model. In this study, the optimal number of LVs was selected according to the basis of including additional factors only when the root mean square error (RMSE) of cross-validation (RMSECV) improved at least 5% [34].

The variable importance in projection (VIP) is a summary of how much a variable contributes to describe the dependent and independent variables in the PLS model [35]. Spectral variables with VIP scores above 1.0 were considered as important contributors for full-spectrum PLS modeling in this study.

### Genetic algorithm combined with partial least squares

Genetic algorithm combined with partial least-squares (GA-PLS) is a variable selection method inspired by natural selection mechanisms [36, 37]. In GA-PLS applied to spectral data, an individual is described as a chromosome that is a combination of genes (spectral variables). The most informative chromosomes are most likely chosen to reproduce and generate offspring through the process of fitness assessment, cross-over, and mutation In this case the GA-PLS was executed on 100 separate occasions, and only subsets selected most frequently by the replicates (greater than 50%) were retained in the final regression model. According to the advice provided by Leardi *et al*. [38], spectral variables were reduced from 467 to 156 using the mean of the absorbance values at 3 contiguous wavenumbers (and the mean of the last 2) to avoid the efficiency of prediction decrease.

### Model evaluation standard

RMSECV was the RMSE calculated from the calibration set (leave-one-out cross-validated samples) and indicated the error of the proposed calibration models. The RMSE of prediction (RMSEP) was calculated from the prediction set to evaluate the prediction ability of different PLS models using the samples of the prediction set. Relative error (RE %) was calculated as the percentage ratio of RMSE (RMSECV or RMSEP) and average value. Also, the ratio RMSEP/RMSECV was adopted to evaluate the robustness of the model. A model with RMSEP/RMSECV lower than 1.2 was usually considered robust [39, 40]. The squared correlation coefficient (*R*^2^) can evaluate the goodness of fitting between actual and predicted values. The closer (*R*^2^) to 1, the better the model fitted.

### Similarity index

As a kind of point-to-point similarity index, Euclidean cosine squared (Cos) was used here for quantitative evaluation of the degree of match of two spectra. The value of Cos was in the interval [0,1], from 0 (no match) to 1 (perfect match).

### Analysis of variance

A one-way analysis of variance and post hoc least significant difference/Tamhane test were used to determine whether *A*_414_ values in UV–Vis spectrum or similarity indices (dependent variable) were significantly different at different PMIs (independent variable). *P* values less than 0.05 were considered statistically significant.

### Software

All data analysis was done on MATLAB R2014a (The MathWorks, MA, USA), Unscrambler Version 9.7 (CAMO Software A/S, Trondheim, Norway), and IBM SPSS Statistics Version 20 (IBM Corporation, NY, USA). GA-PLS was run on Leardi's GA-PLS Toolbox in Matlab [41].

## Result and discussion

### Plasma color inspection and UV-Vis spectroscopic analysis

**Fig 1:**
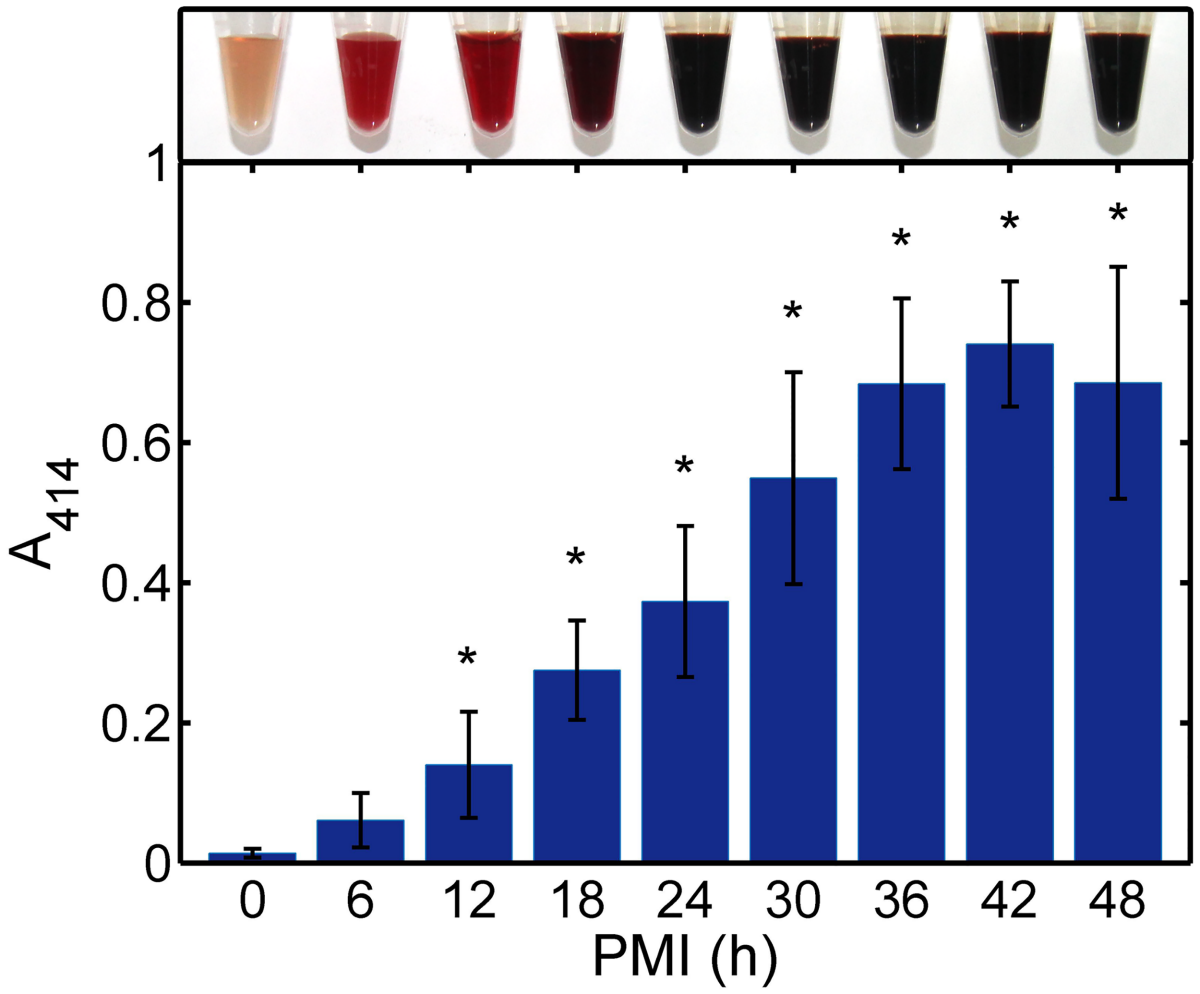
Plasma color inspection and the *A*_414_ values. A typical change in plasma color was compared with the average absorbance measured at 414 nm and the standard deviations. *P < 0.05 indicates a significant difference from the 0-h group.

The plasma exhibited the characteristic discoloration (from pale yellow to pink, and then to dark red with an increase in PMI) after the centrifugation of blood samples (Fig 1). It indicated that hemoglobin, which gave blood red color, might be involved in this postmortem phenomenon. UV-Vis measurement was used to further confirm hemoglobin levels in postmortem plasma samples. It is already known that the main hemoglobin-related peak is located at *λ* = 414 nm and the absorbance at 414 nm (*A*_414_) is correlated with an increase in free hemoglobin concentration [42, 43]. Accordingly, we observed that *A*_414_ values increased significantly with increasing PMI (*P* < 0.05) and decreased slightly at 48 h (Fig 1) showing the accumulation of hemoglobin in the plasma. As hemoglobin is an intracellular protein, the increase in its content in postmortem plasma suggested the presence of the postmortem redistribution of intracellular components caused by the rupturing of blood cells and the release of their contents into the surrounding plasma.

### ATR–FTIR analysis

#### Spectral comparison among plasma of PMI groups

**Fig 2:**
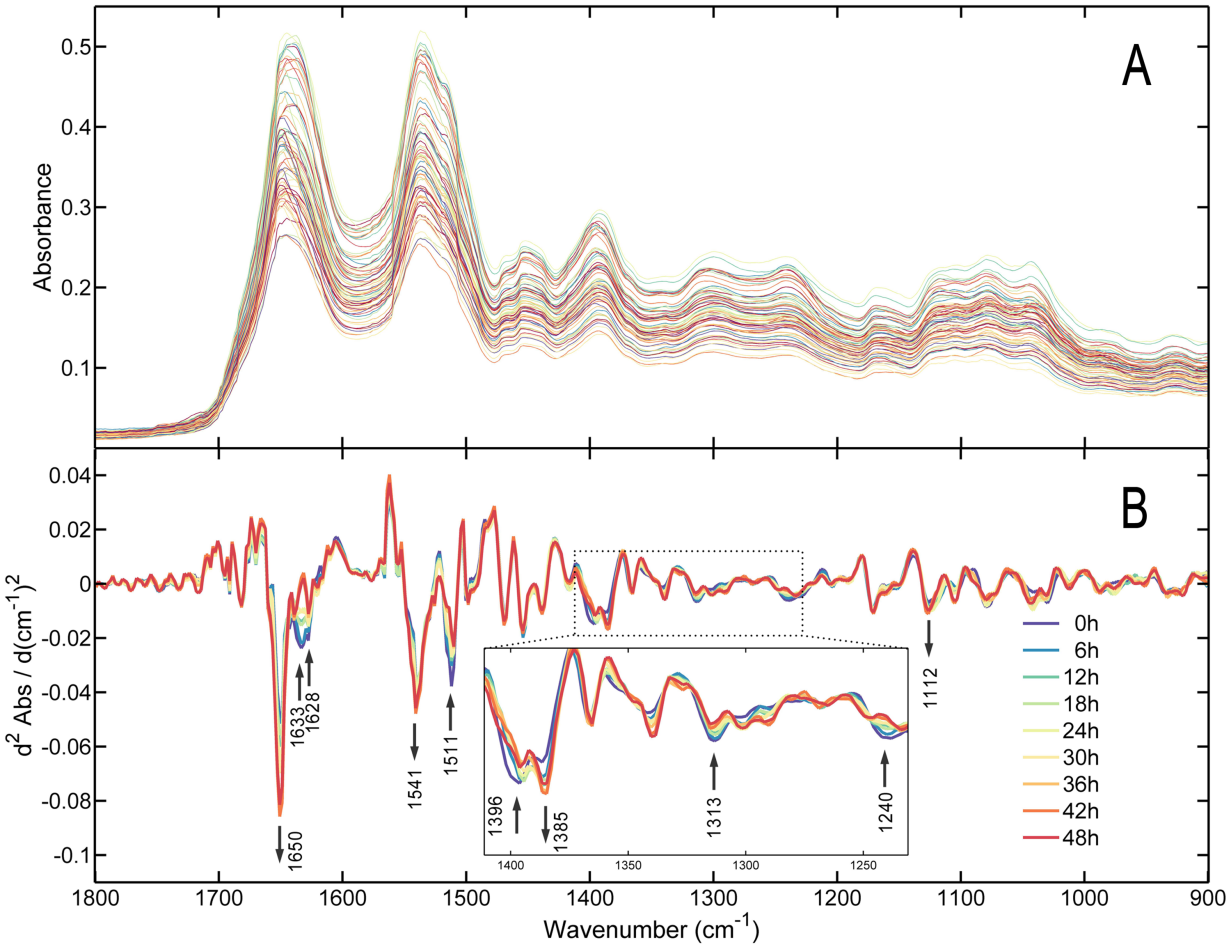
The raw ATR–FTIR spectra and average second-derivative spectra. (A) Raw ATR–FTIR spectra obtained from postmortem plasma. (B) Average SNV-corrected, second-derivative ATR–FTIR spectra of the calibration set plasma obtained from different PMIs. Arrows indicate the major change trends in the intensity of the marked bands with PMI. Inset: local magnification image corresponding to the frame area marked in.

Fig 2A presents the raw ATR–FTIR spectra of the plasma in the range of 1800–900 cm^–1^, and Fig 2B shows the comparison of averaged second-derivative spectra obtained from different PMI groups where the negative peaks corresponded to the center of absorbance peaks of the nonderivative spectrum. Multiple components in the plasma were discriminated simultaneously based on these peaks; their assignment is summarized in Table 1. At first glance, several spectral variances, as revealed by fluctuating intensities, were identified among PMI groups. The most remarkable changing band, observed at 1650 cm^–1^, was related to the α-helix in the Amide I region. Meanwhile, the other two bands at 1633 cm^–1^ and 1628 cm^–1^ in the Amide I region related to the β-sheet also decreased in intensity. Furthermore, the negative peak in the second-derivative spectra at 1633 cm^–1^ gradually disappeared and changed to a positive peak over time. The band at 1541 cm^–1^ represented an increasing tendency, while the bands originating from tyrosine-rich proteins at 1511 cm^–1^ decreased in the Amide II region. The bands in the region between 1480 cm^–1^ and 1430 cm^–1^ displayed the C-H bending (scissoring) vibrations of -CH_2_ and -CH_3_ groups from proteins, phospholipids, and fatty acids [44, 45]. The band at 1396 cm^–1^, which was due to COO^-^ stretching, and the band at 1385 cm^–1^ displayed increase and decrease in intensities, respectively. The band at 1313 cm^–1^ in the Amide III region was blue-shifted to a higher value of 1315 cm^–1^ and decreased. The asymmetric PO_2_^-^ stretching band at 1240 cm^–1^, which was mainly associated with phospholipids, nucleic acids, and phosphate, decreased over time. The band at 1122 cm^–1^ was correlated with the C-O stretching vibrations mode of lactate, whose intensity also increased. The bands at 1080 cm^–1^ and 1040 cm^–1^, which were assigned to symmetric vibration of PO_2_^-^ and COH bending vibration respectively, showed the irregularity in the intensity changes.

**Table 1:** Major band assignments of the FTIR spectrum of plasma and formed elements on the 1800–900 cm^−1^ spectral range [44, 46–48, 67–70].

Wavenumber values (cm^−1^)	Band assignment
**1650**	Amide I: α-helix
**1633–1628**	Amide I: β-sheet
**1541**	Amide II: β-sheet
**1511**	Tyrosine
**1480–1430**	δ_as_(CH_3_), δ_as_(CH_2_), δ_s_(CH_3_), δ_s_(CH_2_): proteins, phospholipids, fatty acids
**1396**	ν_s_(COO^-^): free amino acids
**1315–1313**	Amide III
**1240**	ν_as_(PO_2_^-^): phospholipids, nucleic acids, phosphate
**1126–1122**	ν(C-O): lactate
**1080**	ν_s_(PO_2_^-^): phospholipids, nucleic acids, phosphate, saccharides
**1040**	δ(COH): glucose, polysaccharides

ν, Stretching vibrations (s, symmetric; as, asymmetric); δ, bending (scissoring) vibrations.

#### PLS models based on the FTIR spectral dataset for PMI estimation

**Fig 3:**
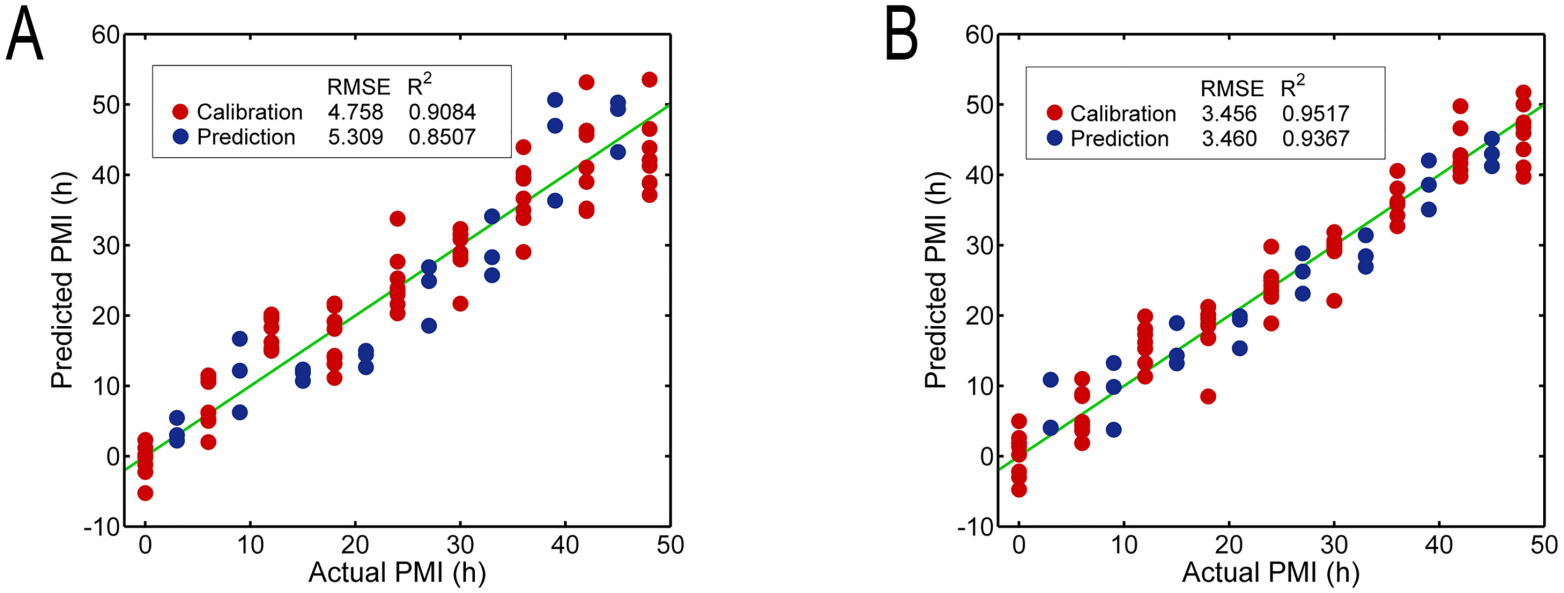
Predicted versus actual PMI from the calibration and prediction sets. (A) Prediction results using the full-spectrum PLS model and (B) using the GA-PLS model. The green lines are the reference line corresponding to the perfect prediction.

Further investigation was performed to explore whether these spectral variances could be used for estimating PMI. Two types of PLS models were constructed based on full-spectrum (spectral range from 1800 to 900 cm^–1^) and GA-selected variables; the detailed results of their performance are summarized in Table 2. In the full-spectrum PLS model, a relatively satisfactory performance was made based on 467 variables with a high *R*^2^ (cross-validation: 0.91, prediction: 0.85) but large error margin of RMSE (RMSECV: 4.76 h, RMSEP: 5.31 h), when three latent factors were determined, explaining 94.82% of total variances. The result is shown in Fig 3A, where most scattering points from different groups were distributed close to the reference line. RMSEP/RMSECV ratio of 1.12 demonstrated the robustness of model predictive ability.

**Table 2:** Results of cross-validation and predictive ability of the full-spectrum model and GA-PLS model.

Method	Variable number	LVs	Cross-validation			Prediction		RMSEP/RMSECV
			*R*^*2*^	RMSECV (RE %)	Explained variance	*R*^*2*^	RMSEP (RE %)	
**Full-spectrum PLS**	467	3	0.91	4.76 (19.90)	94.828	0.85	5.31 (22.12)	1.12
**GA-PLS**	168	6	0.95	3.46 (14.45)	98.147	0.94	3.46 (14.42)	1.00

LVs, Latent variables; R^2^, squared correlation coefficient RE %, relative error (%) in cross-validation or prediction; RMSECV, root mean square error of cross-validation; RMSEP, root mean square error of prediction.

In contrast, the GA-PLS model appeared to be better than the full-spectrum PLS model with a higher *R*^2^ (cross-validation: 0.95, prediction: 0.94) and lower error of RMSE (RMSECV: 3.46 h, RMSEP: 3.46 h), and the ratio RMSEP/RMSECV reduced to 1.0. The results of cross-validation and prediction are presented in Fig 3B. In comparison with the original dataset composed of 467 variables, only 168 spectral variables were selected by GA algorithm to establish the PLS model where the data size was reduced by 64%. It demonstrated that the relatively informative variables were selected, and variables carrying weak useful or even useless information were eliminated by GA-PLS. Although the full-spectrum PLS model provided a desirable prediction for PMI estimation, a large number of uncorrelated or useless variables were included, thus increasing model complexity and computational burden.

**Fig 4:**
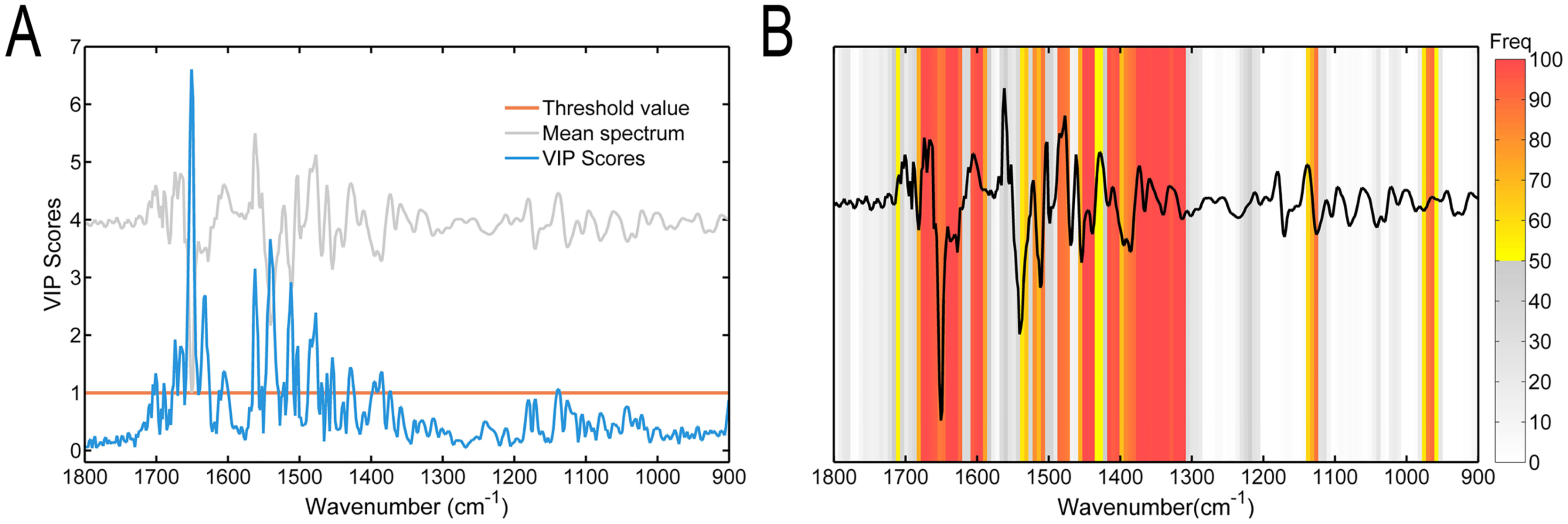
The results of VIP scores and GA-PLS. (A) VIP score distribution for the investigated spectral region 1800–900 cm^–1^. The gray line shows the average preprocessed spectrum, and the orange line shows the threshold value. (B) Frequency of the selection of different wavenumbers in the 100 runs of GA-PLS. The average preprocessed spectrum is superimposed on the frequency.

For information on most informative wavenumbers, the VIP scores of full-spectrum PLS model and the distribution of selected subsets of GA-PLS model are shown in Fig 4A and 4B, respectively. The high VIP scores (above 1.0) in Fig 4A were visible within the spectrum wavenumbers ranging between 1705 and 1373 cm^–1^, which were associated with protein conformations and amino acids. The highest peaks in VIP scores at 1651 cm^–1^, 1633 cm^–1^, 1560 cm^–1^, 1541 cm^–1^, and 1512 cm^–1^ belonged to Amide I and II regions, which were correlated with protein conformation. Wavenumbers in the spectral region 1372–900 cm^–1^ had low VIP scores, which were mainly relevant to the saccharides, lactate, nucleic acids, and phospholipids. Moreover, four unevenly distributed regions had a high degree of stability in the process of GA-PLS (Fig 4B). The first region (1684–1587 cm^–1^) was characteristic of the Amide I bands of proteins. The second region (1539–1309 cm^–1^) was the combination of Amide II bands, the C-H bending (scissoring) vibrations in -CH_2_ and -CH_3_ groups, and the C = O symmetric stretching in COO^-^ group. The third region (1140–1122 cm^–1^) was mainly for the lactate [46–48]. The wavenumbers selected most frequently in the fourth region (975–958 cm^–1^) were not related to any characteristic negative peak of the second-derivative spectra and provided less valuable information. They were selected possibly because they became more important when added to other more specific regions. On the contrary, spectral regions (1308–1141 cm^–1^, 1121–976 cm^–1^, and 957–900 cm^–1^) carrying less useful information were rarely selected by GA-PLS. These regions were mainly related to phospholipids, nucleic acids, and saccharides. As an exception, the region around 1240 cm^–1^ associated with phospholipids and nucleic acids seemed to decrease over time visually, but its contribution to the regression model was judged to be relatively less by GA-PLS.

The two methods of evaluation were different in few aspects. The spectral region from 1417 cm^–1^ to 1309 cm^–1^ selected by GA-PLS mostly had low VIP scores. Moreover, the area around 1541 cm^–1^ with a second highest peak in VIP scores was not selected frequently in GA-PLS. Each spectral wavenumber in the region from 1417 cm^–1^ to 1309 cm^–1^ might become more contributive when collaborated with some other regions, although they might not be important separately in the full-spectrum PLS model. The contrary was the vicinity of 1541 cm^–1^. These differences might be caused by the difference in these two algorithms. VIP scores were proposed to evaluate the importance of each variable (i.e., spectral wavenumber) in the PLS model [49]. However, GA-PLS was a multivariate approach for variable selection that aimed to find out the combination producing the best response [50]. In general, the regions selected mostly by GA-PLS and regions with high VIP scores were largely consistent and might be supported by each other; more spectrum wavenumbers were selected as informative variables by GA-PLS than VIP scores.

#### Spectral comparison between plasma of different PMI groups and formed elements

**Fig 5:**
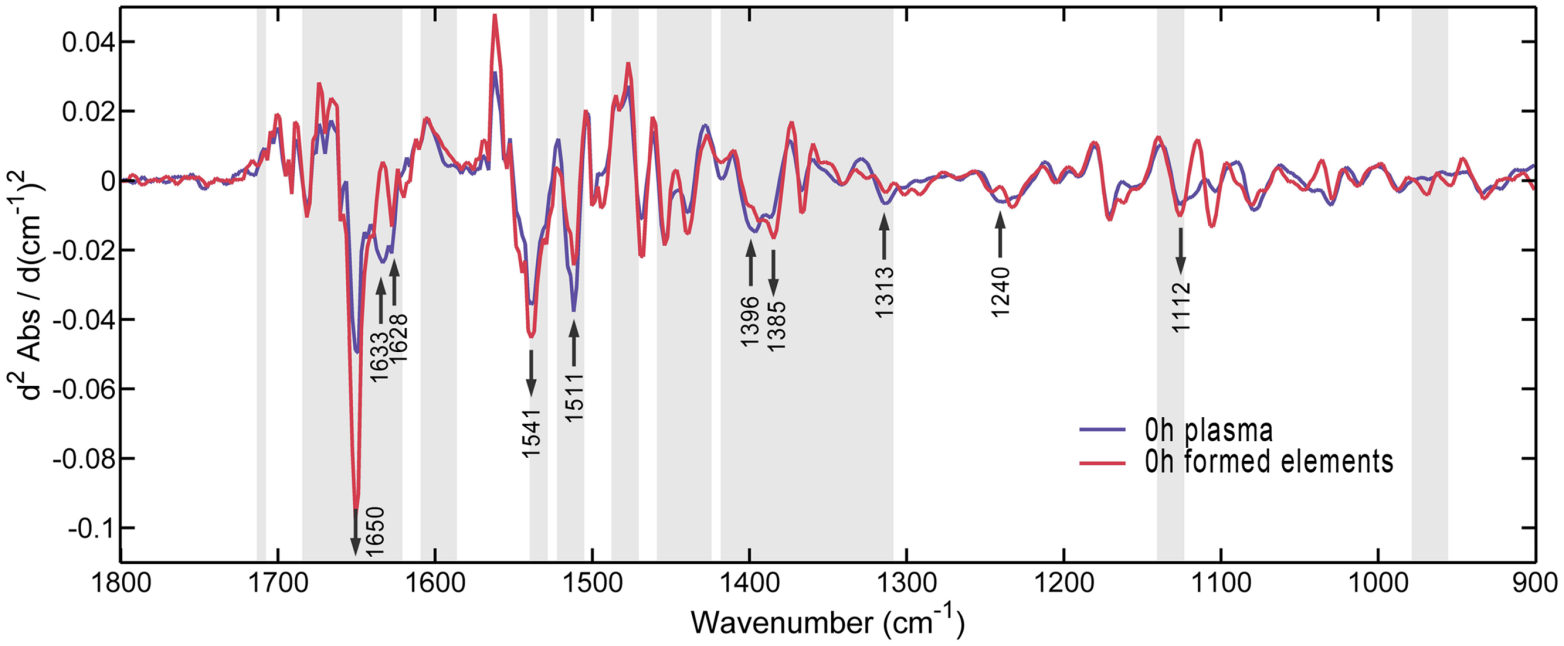
Comparison of spectra of 0-h plasma and formed elements. Arrows denote the spectral differences at corresponding bands in Fig 2B and point the direction from 0-h plasma to formed elements. Informative spectral subsets selected most frequently by GA-PLS (greater than 50%) are represented in gray, and others are in white.

The results of visual inspection and UV–Vis spectroscopy demonstrated the presence of postmortem redistribution. This phenomenon should also affect the changes in FTIR spectrum. It might be expected that if the plasma were mixed with the components from formed elements, the spectrum would become more similar to the latter and the similarity would be closely related to the concentration ratio. Fig 5 shows the averaged second-derivative spectra obtained from plasma and formed elements from the 0-h group and clearly revealed the spectral differences in peak positions and intensities. As expected, these differences visually corresponded with the spectral variances observed in the averaged second-derivative spectra among PMI groups (Fig 2b), especially in the GA-selected regions.

**Fig 6:**
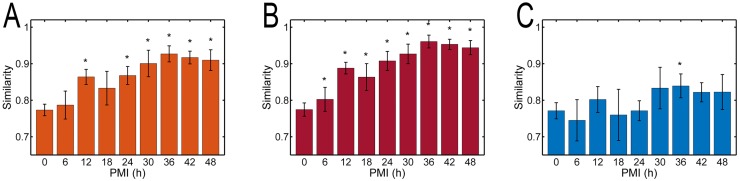
Average similarity index and the standard deviations at each PMI point. The spectra from different PMI plasma groups were compared with the averaged spectrum from 0-h formed elements using (A) full-spectrum subsets, (B) selected subsets, and (C) eliminated subsets by the GA-PLS model. *P < 0.05 indicates a significant difference from the 0-h group.

It is worth highlighting that signal intensity in a second-derivative spectrum is mainly caused by a curvature of the absorbance spectrum rather than the concentration of a certain component. Moreover, visual inspection may reveal some similarities and differences in the spectra, but it is not entirely obvious how the global spectral changes occur. Therefore, the spectral similarity was investigated, where the spectra from different PMI plasma groups were compared with the averaged spectrum from 0-h formed elements to further confirm the main trend of spectral changes. The three regions (full-spectrum, GA-selected, and GA-excluded) were used separately (Fig 6). For the full-spectrum and the GA-selected subsets, the results were similar except that the latter with higher similarity indices and less standard deviations indicating GA indeed screened out most of the sensitive spectral information. Fig 6A and 6B shows that the similarity increased to a maximum at 36 h (*P* < 0.05) and then reached a plateau until 48 h (*P* > 0.05). These results supported the viewpoint that biochemical changes due to redistribution (*i*.*e*., diffusion) of intracellular components, which dominated the main postmortem spectral changes in plasma, were time-independent in the first 36 h. It is worth mentioning that the similarity indices were variable and showed no significant tendency in the GA-excluded regions (Fig 6C), indicating less influence of the redistribution of intracellular components.

#### Explanation of FTIR spectral changes in postmortem plasma

**Fig 7:**
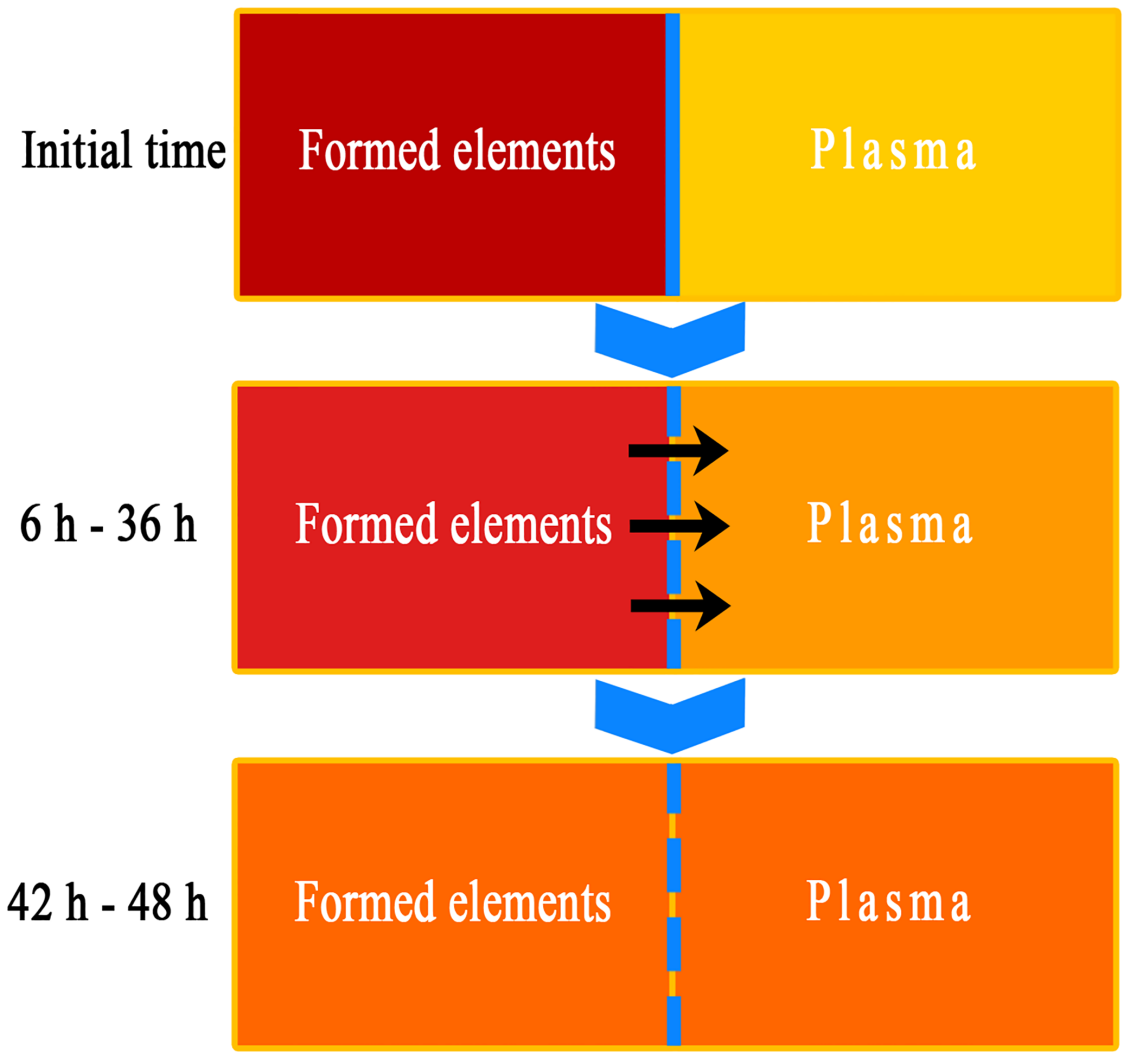
Order and degree of postmortem changes in blood after death as time progresses. The blue lines represent cell membranes (solid lines: intact; dotted line: broken-down), and the black arrows represent the effects of redistribution.

The role of postmortem redistribution in forensic lab examination is often neglected, and even generally considered to disturb the biochemical analyses of blood. Conversely, as this phenomenon affects the contents of surrounding plasma, it may have the potential to become an indicator of postmortem changes. Blood plasma is a pale yellow extracellular fluid that normally holds the formed elements (including the red blood cells, white blood cells, and platelets) in whole blood in suspension. It is mostly water and contains dissolved proteins, glucose, clotting factors, electrolytes, hormones, and gases. In fresh blood, formed elements, which are suspended in the plasma, can be easily separated by centrifugation. The components in formed elements are relatively isolated from the plasma by membranes in a healthy living body. The diffusion due to the breakdown of cell membranes is a result of autolysis [51–53]. Its mechanism is similar to that of the postmortem leakage of potassium from the retina into the center of the globe, which helps estimate the PMI [51]. A scheme has been generated (Fig 7) to demonstrate in a more illustrative way what occurs to postmortem blood. Although the cells in blood are deprived of oxygen after death, the loss of selective membrane permeability, and subsequently the dissolution of cells, eventually causes the release of intracellular rich components into the plasma and surrounding tissue spaces [54], as demonstrated in Fig 7.

As a spectroscopic result, the spectral features of plasma are gradually replaced by the ones of formed elements with the band at 1650 cm^–1^ most typically. Although hemoglobin, an abundant component in formed elements, has no characteristic absorption peak in the infrared spectrum, its infrared spectrum is characterized by a great predominance of α-helix secondary structure absorbance at 1650 cm^–1^ [30, 55, 56]. The increase in intensity at 1650 cm^–1^ showed indirectly the increase in free hemoglobin in postmortem plasma, which was consistent with the results obtained by UV–Vis spectroscopy.

This theory of postmortem redistribution can also explain why the spectral region relevant to proteins, free amino acids, fatty acids, and lactate had high VIP scores and/or was selected mostly by GA-PLS. These components had two common traits: (1) they easily diffuse into the plasma, and (2) corresponding spectral regions have large differences between 0-h plasma and 0-h formed elements. These two traits were both conducive to cause regular changes in corresponding spectral regions.

The regions around 1240 cm^–1^ and 1080 cm^–1^, which were mainly assigned to ν_as_(PO_2_^−^) and ν_s_(PO_2_^−^), respectively, in nucleic acids, phosphate, and phospholipids were selected less frequently by GA-PLS. It demonstrated that the contribution of PO_2_^-^ group to the regression model was relatively small. Nucleic acids are macromolecules mainly in the nucleus of white blood cells. Phospholipids mainly exist in cell membranes, and therefore they cannot easily diffuse into the plasma. Moreover, the saccharide-related spectral regions (1200–900 cm^–1^, but not including the region around 1126 cm^–1^) carrying less sensitive information can also be explained by the characteristics of saccharides. As the main source of saccharides in the blood *in vivo*, glucose diffuses across membranes and into the cells through facilitated diffusion [57]. Therefore, no concentration gradient exists to drive the reverse diffusion of saccharides through the broken cell membranes after death. Furthermore, the concentrations of blood saccharides also fluctuate resulting from food intake, which may also lead to the instability of their postmortem initial values and go against the robustness of the prediction model. Therefore, these spectral regions were little influenced by the postmortem redistribution and contributed little to the PLS models.

It is known that decomposition occurs soon after death involving two processes: autolysis and putrefaction caused by the intracellular enzymes and bacterial intrusion [58–60]. Contrary to the postmortem redistribution that decreases with the decrease in the concentration gradient, the decomposition effects increase gradually in the early postmortem period till at some time point, the previously hidden effect of decomposition begins to appear. According to previous researches [22, 61–63], the process of decomposition may also degrade the components in blood, including metabolites, proteins, saccharides, and nucleic acids. Previous studies also proved that the decomposition process had a significant impact on the FTIR spectra of postmortem substantial organs [24, 25, 27]. In this case, the band at 1126 cm^–1^ was associated with lactate and selected as an informative region by GA-PLS. The 1126 cm^–1^ band increased over time; the possible mechanism was that, as a product of anaerobic glycolysis, lactate accumulated in red blood cells rapidly after death and then gradually diffused into the plasma. Unlike the proteins, free amino acids, and fatty acids, this was a typical result of autolysis-induced diffusion combined with postmortem metabolism. This result was supported in part by a previous finding that the lactate concentration in the plasma increased significantly after death [23, 64–66]. The intensities of almost all the other bands in GA-selected regions changed to the levels of the corresponding ones in the spectrum of 0-h formed elements, and their variations could be explained by the phenomenon of postmortem decomposition. If the components in postmortem plasma (including the part from formed elements) decomposed significantly to drive the spectral changes at these time points, the similarity indices (compared with the 0-h formed elements) would decrease accordingly. Although the similarity indices in both full-spectrum and GA-selected regions increased in the first 36 h and then remained almost unchanged (Fig 6A and 6B), the decrease in average similarity index at 42 and 48 h was negligible (*P* > 0.05). This result could only indicate that the postmortem redistribution faded away and the system changed to a relative equilibrium state at 36 h. As no samples were taken beyond the 48-h time interval, it was not suitable to judge any statistically significant decline in similarity index after this time point. However, it could be proved at least that the effect of decomposition in the plasma was masked greatly by the strong effect of postmortem redistribution in spectroscopy within postmortem 48 h.

In general, the effects of redistribution on postmortem plasma in 48 h were observed. The effect of redistribution dominated the FTIR spectral changes and masked the effect of decomposition, especially in 36 h after death. This process of PMI-dependent redistribution existed only after death, and would not occur due to vital/postmortem degradation (metabolic process) and redistribution, which was close to the ideal model expected by Claus Henssge and Burkhard Madea [8]. According to Burkhard Madea [51], this postmortem process of redistribution in blood was also a suitable analyte for estimating the PMI because of the high concentration gradient between plasma and formed element components, especially the level of hemoglobin (hemoglobin normally accounts for about 90% of erythrocyte proteins and almost 0% of plasma proteins) [45].

## Conclusions

In summary, ATR-FTIR spectroscopy achieved the purpose of estimating PMI and monitoring the postmortem molecular changes using the postmortem arterial blood plasma. By establishing PLS models for PMI estimation, the results showed a satisfactory predictive ability on the whole with the best RMSECV of 3.46 h (*R*^*2*^ = 0.95) and RMSEP of 3.46 h (*R*^*2*^ = 0.94). Moreover, spectral regions related to proteins, free amino acids, fatty acids, and lactate were identified with regular changes and played an important role in estimating PMI. Furthermore, the results showed that the redistribution of intracellular components dominated the spectral changes in postmortem plasma especially in the first 36 h, and the decomposition seemed to contribute little to the changes. However, these results were valid only under constant environmental conditions. The finding of this study might help to better understand the redistribution of postmortem components in blood and provide a new way to estimate the PMI. Further studies should be conducted to explore the potential effect of changes in temperature, humidity, and so forth on the PMI to meet the requirement of forensic application.

## Supporting information

S1 Dataset Spectral data. The raw ATR–FTIR spectral data of postmortem rabbit plasma.
(XLSX)

S2 Dataset UV-Vis spectral data. The absorbance values of UV-Vis spectra at 414 nm.
(XLSX)

## References

[1] Kikuchi K, Kawahara KI, Biswas KK, Ito T, Tancharoen S, Shiomi N, et al HMGB1: A new marker for estimation of the postmortem interval. Exp Ther Med. 2010;1(1):109–11. doi: 10.3892/etm_00000019 23136602 10.3892/etm_00000019 PMC3490379

[2] Poloz YO, O'day DH. Determining time of death: temperature-dependent postmortem changes in calcineurin A, MARCKS, CaMKII, and protein phosphatase 2A in mouse. Int J Legal Med. 2009;123(4):305–14. doi: 10.1007/s00414-009-0343-x 19326139 10.1007/s00414-009-0343-x

[3] Kurtulus A, Acar K, Sorkun H, Kelten C, Boz B. The relationship between adrenal gland morphometric changes and postmortem interval in rats: A stereological study. Leg Med. 2012;14(4):214–8. doi: 10.1016/j.legalmed.2012.03.002 22503244 10.1016/j.legalmed.2012.03.002

[4] Machaalani R, Gozal E, Berger F, Waters KA, Dematteis M. Effects of post-mortem intervals on regional brain protein profiles in rats using SELDI-TOF-MS analysis. Neurochem Int. 2010;57(6):655–61. doi: 10.1016/j.neuint.2010.08.002 20708053 10.1016/j.neuint.2010.08.002

[5] Warther S, Sehner S, Raupach T, Puschel K, Anders S. Estimation of the time since death: post-mortem contractions of human skeletal muscles following mechanical stimulation (idiomuscular contraction). Int J Legal Med. 2012;126(3):399–405. doi: 10.1007/s00414-011-0665-3 22245837 10.1007/s00414-011-0665-3

[6] Mao S, Dong X, Feng F, Seese RR, Wang Z. Estimation of postmortem interval using an electric impedance spectroscopy technique: a preliminary study. Sci Justice. 2011;51(51):135–8. 21889110 10.1016/j.scijus.2010.11.003

[7] Mao S, Fu G, Seese RR, Wang ZY. Estimation of PMI depends on the changes in ATP and its degradation products. Leg Med. 2013;15(5):235–8. 10.1016/j.legalmed.2013.03.004 23639682

[8] Henssge C, Madea B. Estimation of the time since death in the early post-mortem period. Forensic Sci Int. 2004;144(2–3):167–75. doi: 10.1016/j.forsciint.2004.04.051 15364387 10.1016/j.forsciint.2004.04.051

[9] Amendt J, Campobasso CP, Gaudry E, Reiter C, LeBlanc HN, Hall MJR. Best practice in forensic entomology—standards and guidelines. Int J Legal Med. 2007;121(2):90–104. doi: 10.1007/s00414-006-0086-x 16633812 10.1007/s00414-006-0086-x

[10] Sowemimo-Coker SO. Red blood cell hemolysis during processing. Transfus Med Rev. 2002;16(1):46–60. doi: 10.1053/tmrv.2002.29404 11788929 10.1053/tmrv.2002.29404

[11] Appierto V, Callari M, Cavadini E, Morelli D, Daidone MG, Tiberio P. A lipemia-independent NanoDrop(^®^)-based score to identify hemolysis in plasma and serum samples. Bioanalysis. 2014;6(9):1215–26. doi: 10.4155/bio.13.344 24946922 10.4155/bio.13.344

[12] Baker MJ, Trevisan J, Bassan P, Bhargava R, Butler HJ, Dorling KM, et al Using Fourier transform IR spectroscopy to analyze biological materials. Nat Protoc. 2014;9(8):1771–91. doi: 10.1038/nprot.2014.110 24992094 10.1038/nprot.2014.110 PMC4480339

[13] Movasaghi Z, Rehman S, Rehman IU. Fourier transform infrared (FTIR) spectroscopy of biological tissues. Appl Spectrosc Rev. 2008;43(2):134–79. doi: 10.1080/05704920701829043

[14] Jiang C, Liu J, Rubacha M, Shukla AA. A mechanistic study of Protein A chromatography resin lifetime. J Chromatogr A. 2009;1216(31):5849–55. doi: 10.1016/j.chroma.2009.06.013 19539295 10.1016/j.chroma.2009.06.013

[15] Barth A. Infrared spectroscopy of proteins. Biochim Biophys Acta. 2007;1767(9):1073–101. doi: 10.1016/j.bbabio.2007.06.004 . 17692815 10.1016/j.bbabio.2007.06.004

[16] Mukhopadhyay R. Portable FTIR spectrometers get moving. Anal Chem. 2004;76(19):369a–72a. doi: 10.1021/Ac041652z 15487052 10.1021/ac041652z

[17] Rein AJ, Seelenbinder J. Handheld and Portable FTIR Spectrometers for the Analysis of Materials: Taking the Lab to the Sample. Am Lab. 2013;45(6):16–9.

[18] Donaldson AE, Lamont IL. Estimation of post-mortem interval using biochemical markers. Aust J Forensic Sci. 2014;46(1):8–26. doi: 10.1080/00450618.2013.784356

[19] Schoning P, Strafuss AC. Determining Time Of Death Of a Dog by Analyzing Blood, Cerebrospinal-Fluid, And Vitreous-Humor Collected at Postmortem. Am J Vet Res. 1980;41(6):955–7. 7436089

[20] Dokgoz H, Arican N, Elmas I, Fincanci SK. Comparison of morphological changes in white blood cells after death and in vitro storage of blood for the estimation of postmortem interval. Forensic Sci Int. 2001;124(1):25–31. doi: 10.1016/S0379-0738(01)00559-X 11741756 10.1016/s0379-0738(01)00559-x

[21] Costa I, Carvalho F, Magalhaes T, de Pinho PG, Silvestre R, Dinis-Oliveira RJ. Promising blood-derived biomarkers for estimation of the postmortem interval. Toxicol Res-Uk. 2015;4(6):1443–52. doi: 10.1039/c5tx00209e

[22] Donaldson AE, Lamont IL. Metabolomics of post-mortem blood: identifying potential markers of post-mortem interval. Metabolomics. 2015;11(1):237–45.

[23] Sato T, Zaitsu K, Tsuboi K, Nomura M, Kusano M, Shima N, et al A preliminary study on postmortem interval estimation of suffocated rats by GC-MS/MS-based plasma metabolic profiling. Anal Bioanal Chem. 2015;407(13):3659–65. doi: 10.1007/s00216-015-8584-7 25749795 10.1007/s00216-015-8584-7

[24] Tuo Y, Huang P, Ke Y, Fan SL, Lu QY, Xin B, et al Attenuated Total Reflection Fourier Transform Infrared Spectroscopic Investigation of the Postmortem Metabolic Process in Rat and Human Kidney Cortex. Appl Spectrosc. 2010;64(3):268–74. doi: 10.1366/000370210790918382 20223060 10.1366/000370210790918382

[25] Huang P, Ke Y, Lu QY, Xin B, Fan SL, Yang GD, et al Analysis of postmortem metabolic changes in rat kidney cortex using Fourier transform infrared spectroscopy. Spectrosc-Int J. 2008;22(1):21–31. doi: 10.3233/Spe-2008-0340

[26] Huang P, Tian W, Tuo Y, Wang Z, Yang G. Estimation of Postmortem Interval in Rat Liver and Spleen Using Fourier Transform Infrared Spectroscopy. Spectrosc Lett. 2009;42(2):108–16.

[27] Yong K, Yang L, Wang ZY. The Changes of Fourier Transform Infrared Spectrum in Rat Brain *. J Forensic Sci. 2012;57(3):794–8. doi: 10.1111/j.1556-4029.2011.02036.x 22221050 10.1111/j.1556-4029.2011.02036.x

[28] Martin FL, Kelly JG, Llabjani V, Martinhirsch PL, Patel II, Trevisan J, et al Distinguishing cell types or populations based on the computational analysis of their infrared spectra. Nat Protoc. 2010;5(11):1748–60. doi: 10.1038/nprot.2010.133 21030951 10.1038/nprot.2010.133

[29] Rinnan Å, Berg FVD, Engelsen SB. Review of the most common pre-processing techniques for near-infrared spectra. TrAC, Trends Anal Chem. 2009;28(10):1201–22.

[30] Yang H, Yang S, Kong J, Dong A, Yu S. Obtaining information about protein secondary structures in aqueous solution using Fourier transform IR spectroscopy. Nat Protoc. 2015;10(3):382–96. doi: 10.1038/nprot.2015.024 25654756 10.1038/nprot.2015.024

[31] Ziegel ER. A user-friendly guide to multivariate calibration and classification. Technometrics. 2002;17(1):108–10.

[32] Mehmood T, Ahmed B. The diversity in the applications of partial least squares: an overview. J Chemom. 2015;30(1):4–17.

[33] Wold S, Sjöström M, Eriksson L. PLS-regression: a basic tool of chemometrics. Chemometrics & Intelligent Laboratory Systems. 2001;58(2):109–30.

[34] Brás LP, Lopes M, Ferreira AP, Menezes JC. A bootstrap-based strategy for spectral interval selection in PLS regression. J Chemom. 2008;22(11–12):695–700.

[35] Andersen CM, Bro R. Variable selection in regression—a tutorial. J Chemom. 2010;24(11–12):728–37.

[36] Leardi R. Application of genetic algorithm–PLS for feature selection in spectral data sets. J Chemom. 2000;14(5–6):643–55.

[37] Hasegawa K, Funatsu K. GA strategy for variable selection in QSAR studies: GAPLS and D-optimal designs for predictive QSAR model. Journal of Molecular Structure Theochem. 1998;425(3):255–62.

[38] Leardi, R. Manual of the PLS-Genetic Algorithm Toolbox. http://www.models.life.ku.dk/sites/default/files/mangapls.pdf, 2004 (accessed 15.03.15).

[39] Coûteaux MM, Sarmiento L, Hervé D, Acevedo D. Determination of water-soluble and total extracTab. polyphenolics in biomass, necromass and decomposing plant material using near-infrared respectroscopy (NIRS). Soil Biol Biochem. 2005;37(4):795–9.

[40] Alves A, Santos A, Rozenberg P, Pâques LE, Charpentier JP, Schwanninger M, et al A common near infrared—based partial least squares regression model for the prediction of wood density of Pinus pinaster and Larix × eurolepis. Wood Science & Technology. 2012;46(1–3):157–75.

[41] Leardi, R. The PLS-genetic algorithm toolbox for Matlab (TM). http://www.models.life.ku.dk/GAPLS, 2004 (accessed 17.03.15).

[42] Kirschner MB, Kao SC, Edelman JJ, Armstrong NJ, Vallely MP, Van ZN, et al Haemolysis during sample preparation alters microRNA content of plasma. PLoS One. 2011;6(9):270–2. 10.1371/journal.pone.0024145 PMC3164711 21909417

[43] Harboe M. A method for determination of hemoglobin in plasma by near-ultraviolet spectrophotometry. Scand J Clin Lab Invest. 1959;11(1):66–70. 13646603 10.3109/00365515909060410

[44] Petibois C, Déléris G. Analysis and monitoring of oxidative stress in exercise and training by FTIR spectrometry. Int J Sports Physiol Perform. 2008;3(2):119–30. 19208921 10.1123/ijspp.3.2.119

[45] Petibois C, Déléris G. Oxidative stress effects on erythrocytes determined by FT-IR spectrometry. Analyst. 2004;129(129):912–6. 15457322 10.1039/b408931f

[46] Petibois C, Déléris G. Evidence that erythrocytes are highly susceptible to exercise oxidative stress: FT-IR spectrometric studies at the molecular level. Cell Biol Int. 2005;29(8):709–16. doi: 10.1016/j.cellbi.2005.04.007 15953739 10.1016/j.cellbi.2005.04.007

[47] Petibois C, Gionnet K, Gonçalves M, Perromat A, Moenner M, Déléris G. Analytical performances of FT-IR spectrometry and imaging for concentration measurements within biological fluids, cells, and tissues. Analyst. 2006;131(5):640–7. doi: 10.1039/b518076g 16633577 10.1039/b518076g

[48] Petibois C, Melin AM, Perromat A, Cazorla G, Déléris G. Glucose and lactate concentration determination on single microsamples by Fourier-transform infrared spectroscopy. J Lab Clin Med. 2000;135(2):210–5. doi: 10.1067/mlc.2000.104460 10695667 10.1067/mlc.2000.104460

[49] Oussama A, Elabadi F, Platikanov S, Kzaiber F, Tauler R. Detection of Olive Oil Adulteration Using FT-IR Spectroscopy and PLS with Variable Importance of Projection (VIP) Scores. J Am Oil Chem Soc. 2012;89(10):1807–12.

[50] Leardi R, González AL. Genetic algorithms applied to feature selection in PLS regression: how and when to use them. Chemometrics & Intelligent Laboratory Systems. 1998;41(2):195–207.

[51] Madea B. Is there recent progress in the estimation of the postmortem interval by means of thanatochemistry? Forensic Sci Int. 2005;151(151):139–49. 15939145 10.1016/j.forsciint.2005.01.013

[52] Gall JAM. Forensic Medicine: Clinical and Pathological Aspects. J R Soc Med. 2003;96(10): 556.14594969

[53] Madea B. Importance of supravitality in forensic medicine. Forensic Sci Int. 1994;69(3):221–41. 7860008 10.1016/0379-0738(94)90386-7

[54] Vass AA, Barshick SA, Sega G, Caton J, Skeen JT, Love JC, et al Decomposition chemistry of human remains: a new methodology for determining the postmortem interval. J Forensic Sci. 2002;47(3):542–53. 12051334

[55] Lee DC, Haris PI, Chapman D, Mitchell RC. Determination of protein secondary structure using factor analysis of infrared spectra. Biochemistry. 1990;29(39):9185–93. 2271587 10.1021/bi00491a012

[56] Severcan M, Severcan F, Haris PI. Estimation of protein secondary structure from FTIR spectra using neural networks. J Mol Struct. 2001;565(2):383–7.

[57] Carruthers A. Facilitated diffusion of glucose. Physiol Rev. 1990;70(4):1135–76. 2217557 10.1152/physrev.1990.70.4.1135

[58] Henssge C, Madea B. Estimation of the time since death. Forensic Sci Int. 2007;165(2–3):182–4. doi: 10.1016/j.forsciint.2006.05.017 16797901 10.1016/j.forsciint.2006.05.017

[59] Hyde ER, Haarmann DP, Petrosino JF, Lynne AM, Bucheli SR. Initial insights into bacterial succession during human decomposition. Dtsch Z Gesamte Gerichtl Med. 2014;129(3):1–11. 10.1007/s00414-014-1128-4 25431049

[60] Gümü f x M.D. A, Gümü f x M.D B, et al Evaluation of the Postmortem Glucose and Glycogen Levels in Hepatic, Renal, Muscle, and Brain Tissues: Is It Possible to Estimate Postmortem Interval Using These Parameters? J Forensic Sci. 2015. 10.1111/1556-4029.12937 26305623

[61] Maeda H. Forensic biochemistry for functional investigation of death: concept and practical application. Leg Med. 2011;13(2):55–67. 10.1016/j.legalmed.2010.12.005 21269863

[62] Bauer M, Gramlich I, Polzin S, Patzelt D. Quantification of mRNA degradation as possible indicator of postmortem interval—a pilot study. Leg Med. 2003;5(4):220–7. 10.1016/j.legalmed.2003.08.001 14602165

[63] Donaldson AE, Lamont IL. Estimation of post-mortem interval using biochemical markers. Aust J Forensic Sci. 2013;46(1):1–19.

[64] Jetter WW, McLean R. Biochemical changes in body fluids after death. Am J Clin Pathol. 1943;13(4):178–85.

[65] Katsumata Y, Sato K, Yada S, Uematsu T, Oya M, Yoshino M. Anaerobic metabolism in dogs after organismal death. Nihon hōigaku zasshi = The Japanese journal of legal medicine. 1983;37(1):75–8. 6676512

[66] Donaldson AE, Lamont IL. Biochemistry Changes That Occur after Death: Potential Markers for Determining Post-Mortem Interval. PLoS One. 2013;8(11):995–8. 10.1371/journal.pone.0082011 PMC3836773 24278469

[67] Staniszewska E, Malek K, Baranska M. Rapid approach to analyze biochemical variation in rat organs by ATR FTIR spectroscopy. Spectrochimica Acta Part A Molecular & Biomolecular Spectroscopy. 2014;118(118C):981–6. 10.1016/j.saa.2013.09.131 24161861

[68] Olsztyńska-Janus S, Szymborska-Małek K, Gąsior-Głogowska M, Walski T, Komorowska M, Witkiewicz W, et al Spectroscopic techniques in the study of human tissues and their components. Part I: IR spectroscopy. Acta Bioeng Biomech. 2012;14:101–15. 23140221

[69] Staniszewska-Slezak E, Fedorowicz A, Kramkowski K, Leszczynska A, Chlopicki S, Baranska M, et al Plasma biomarkers of pulmonary hypertension identified by Fourier transform infrared spectroscopy and principal component analysis. Analyst. 2015;140(7):2273–9. doi: 10.1039/c4an01864h 25599976 10.1039/c4an01864h

[70] Elkins KM. Rapid Presumptive “Fingerprinting” of Body Fluids and Materials by ATR FT-IR Spectroscopy. J Forensic Sci. 2011;56(6):1580–7. doi: 10.1111/j.1556-4029.2011.01870.x 21827466 10.1111/j.1556-4029.2011.01870.x

